# Subsidence of the BFX standard stem after canine total hip replacement: A case series of 177 consecutive procedures

**DOI:** 10.1111/vsu.70084

**Published:** 2026-02-09

**Authors:** Daniel Low, Rhys Treharne, Scott Rutherford

**Affiliations:** ^1^ frank. Pet Surgeons Leeds UK; ^2^ Swift Referrals Wetherby UK

## Abstract

**Objective:**

To describe postoperative subsidence observed after total hip replacement (THR) with the BFX standard stem.

**Study design:**

Retrospective case series.

**Sample population:**

A total of 177 THR procedures in 145 dogs.

**Methods:**

Clinical and radiographic follow‐up data were collected between 12 weeks and six months postoperatively. A proportion correction was used to calculate subsidence between immediate postoperative and follow‐up radiographs. Canal flare index, coronal canal fill, sagittal canal fill, coronal stem orientation and sagittal stem orientation were measured.

**Results:**

There were 24/177 (13.6%) intra‐ and postoperative complications. Of these, 11/177 (6.2%) were stem complications. Eight stem complications were intraoperative femoral fissures; one fissure progressed to postoperative femoral fracture. Subsidence was associated with stem complications (*p* = .005) and was not associated with non‐stem complications (*p* = .76) or prosthetic luxation (*p* = .07). There were seven stems with more than 5.0 mm of subsidence; of these only 1/7 had a stem complication. There were 26 stems with more than 3.0 mm of subsidence; of these only 3/26 had a stem complication. There was no association between any femoral or stem measurements and subsidence.

**Conclusion:**

Subsidence of the BFX standard stem was associated with stem complications, which were mostly intraoperative femoral fissures. Thresholds of 3.0 and 5.0 mm were poorly predictive of complications, and substantial subsidence was often clinically inconsequential.

**Clinical significance:**

Cutoffs for excessive subsidence may have limited clinical utility. The significance of postoperative subsidence should be interpreted in the context of other clinical findings.

## 1 INTRODUCTION

Total hip replacement (THR) is an established surgical procedure for treating canine hip dysplasia^1^ and is the only surgical procedure consistently returning dogs to normal function.^2^, ^3^ Early postoperative complication rates between 8.2% and 9.4%, respectively are reported.^4^, ^5^, ^6^ Cemented implants are at risk of short‐ or long‐term aseptic loosening^7^, ^8^ and cementless implants may be preferred.^9^ The BioMedtrix (Whippany, New Jersey) system with a cementless biologic fixation (BFX) femoral stem is one of the most commonly used cementless THR systems.^4^ Immediate stability of the femoral stem is achieved through press‐fit and friction at the bone‐implant interface.^10^

Early postoperative subsidence of the BFX stem has been reported, which usually occurs within five weeks of initial surgery.^9^ Subsidence has been suggested to be associated with an increased risk of postoperative complications and these concerns are the basis for the BFX collared and BFX lateral bolt stems.^11^, ^12^, ^13^ However, the association between subsidence and complications are unclear, as large subsidence values without complication have been documented with various BFX stems.^13^, ^14^ A small degree of subsidence is expected with a cementless femoral stem, and may even be desirable to improve the press‐fit of the implant.^9^ Subsidence is typically measured on craniocaudal radiographs of the femur but this method is sensitive to radiographic positioning, meaning small subsidence values may be difficult to detect. Therefore, there is no consensus about the definition of excessive subsidence, with suggested cutoffs of 3 and 5 mm proposed.^9^, ^13^

In THR in humans, subsidence is clinically insignificant even when significant subsidence of more than 5 mm occurs, with a 0.4% rate of revision surgery attributable to subsidence.^15^, ^16^ While the BFX lateral bolt stem has been shown to better resist subsidence than the BFX standard stem in an ex vivo study, the implications of this in vivo are unclear.^17^ Subsidence of greater than 3.0 mm of the BFX standard stem has been reported to increase the risk of postoperative stem complications,^18^ however another study did not identify an increased risk of complications despite the BFX standard stem developing greater subsidence than the BFX collared and BFX lateral bolt stems.^13^

This study aimed to describe a case series of dogs undergoing THR with a BFX standard stem, the postoperative subsidence observed, and their association with intraoperative and postoperative complications. The null hypothesis tested was that there would be no association between complications and postoperative subsidence values in BFX standard stems.

## 2 MATERIALS AND METHODS

### 2.1 Data extraction

Retrospective data extraction was performed via hospital electronic health records search (EasyVet; VetZ GmbH) from a single veterinary referral hospital in the UK. All data in this study were obtained with written client consent granting permission for the anonymized use of patient data for research purposes. Records from 2018 to 2025 were searched for “total hip replacement” and “BFX stem” for dogs who underwent THR with a BFX standard stem. Records were excluded if short‐term orthopedic follow‐up examination and radiographs were unavailable, or if follow‐up radiographs were inadequately positioned such that femoral and stem measurements could not be performed. A minimum of 12 weeks and up to six months of postoperative follow‐up data was included in this study. Dogs undergoing bilateral surgery underwent staged bilateral surgery and each limb was considered independently for analysis of subsidence.

### 2.2 Surgical procedure and radiographic evaluation

All THR procedures were performed by a single board‐certified surgeon (European College of Veterinary Surgeons). Preoperative radiographs were obtained for templating and THR surgery was performed in a standard manner^19^ without intraoperative fluoroscopic guidance. Immediate postoperative radiographs were obtained to confirm implant positioning. Dogs were hospitalized postoperatively for 24 h for opioid analgesia. Dogs were discharged with four weeks of a licensed non‐steroidal anti‐inflammatory drug and two weeks of amoxicillin‐clavulanic acid 12.5 mg/kg twice daily. Four weeks of restricted activity was recommended and a gradually increasing regimen of lead‐only walks was introduced from the four‐week follow‐up orthopedic examination. Follow‐up orthopedic examination was performed at four and 12 weeks postoperatively, and follow‐up radiographs obtained routinely at 12 weeks postoperatively. All postoperative complications within six months of THR were re‐examined and treated free‐of‐charge at the same referral hospital, with additional radiographs obtained as necessary in addition to routine follow‐up radiographs. Complications were classified as minor, major, and catastrophic according to proposed definitions.^20^ Complications were subgrouped as stem complications and non‐stem complications.^18^ Stem complications were defined as stem‐related intra‐ or postoperative complications requiring surgical treatment and included intraoperative femoral fissures or fractures and postoperative femoral fractures. Postoperative prosthetic luxation was classified as a stem complication only if there was no identifiable problem with the cup.

Clinical variables collected included breed, sex and neuter status, bodyweight, age at the time of surgery, reason for surgery, laterality, intra‐ and postoperative complications, and implant sizes. Radiographic measurements were obtained on the integrated PACS and DICOM viewer within the hospital electronic health record system. Femoral and stem measurements were obtained on the craniocaudal and mediolateral femur views. Radiographs were calibrated with either a 100 mm bar. Radiographic measurements were made by either one of two observers in an unblinded manner. Radiographic variables included immediate postoperative and follow‐up corrected femoral stem position as previously described,^21^ canal flare index (CFI), canal fill in the coronal (CF_cor_) and sagittal (CF_sag_) planes, and stem orientation in the coronal (SO_cor_) and sagittal (SO_sag_) planes. Subsidence was calculated as the difference between corrected femoral stem position between immediate postoperative radiographs and follow‐up radiographs. Canal flare index was measured on the craniocaudal view of the femur and calculated as a ratio of endosteal width at the lesser trochanter over endosteal width at the narrowest point of the femoral shaft.^22^ Canal fill was calculated as a ratio of implant width to endosteal width at the level of the lateral smooth‐porous junction of the BFX standard stem, 5.0 mm proximal to the distal tip of the femoral stem, and midway between these two points.^18^ Stem orientation was defined as previous, with varus‐oriented stems having a positive SO_cor_ and proximal stem directed cranially having a positive SO_sag_.^23^ For stems with subsidence of more than 3.0 mm, stem version was assessed on follow‐up radiographs and stems which rotated into retroversion were recorded. Version was measured as previously described.^14^

### 2.3 Statistical analysis

Categorical variables are reported descriptively. Data normality of continuous variables was assessed with the Shapiro–Wilk test. Nonparametric data are expressed as median and interquartile range (IQR) and parametric data expressed as mean and SD. As the primary outcome of interest, the range of subsidence values were additionally reported. Femoral and stem measurements were compared between the subgroup of dogs with a stem complication and those without a stem complication, and between the subgroup of dogs with a non‐stem complication and those without a non‐stem complication. Parametrically distributed data were compared with Welch's *t*‐test and nonparametrically distributed data were compared with the Mann–Whitney U test. To evaluate the diagnostic performance of subsidence as a predictor of stem complications, receiver operating characteristic‐based analyses were performed. Subsidence values were used as a continuous test variable, and the presence of a stem complication was used as the binary outcome to calculate Youden's J‐statistic. Statistical significance was defined as *p* < .05. All statistical analysis and data visualization were performed with pandas 2.2.2, numpy 2.0.2, scipy 1.16.1, scikit‐learn 1.6.1, seaborn 0.13.2, and matplotlib 3.10.0 in Python version 3.11.13.^24^, ^25^, ^26^, ^27^, ^28^, ^29^

## 3 RESULTS

### 3.1 Demographic data

A total of 190 consecutive THR procedures with the BFX standard stem in 158 dogs were identified through the database search. There were no THR procedures with the BFX lateral bolt or BFX collared stem. Out of all dogs meeting the minimum size and weight requirement for a BFX stem, two dogs (2 THR procedures) were selected to receive a cemented fixation (CFX) stem. The reason for using a CFX stem was because of a femoral deformity in one dog and because of the contralateral THR with the BFX standard stem experiencing significant subsidence of 9.8 mm in the other dog. This BFX standard stem is included in the final sample population. A total of 10 dogs (10 THR procedures) were excluded as no follow‐up radiographs were available. Two dogs (2 THR procedures) were excluded due to experiencing postoperative femoral fracture and owner‐elected amputation prior to the 12‐week follow‐up. In these two dogs, subsidence values of .4 mm and .8 mm were recorded at the last radiographic evaluation prior to amputation. One dog (one THR procedure) was excluded due to experiencing prosthetic luxation and owner‐elected explantation prior to the 12‐week follow‐up. In this dog, a subsidence value of 0 mm was recorded at the last radiographic evaluation prior to explantation. The sample population consisted of 177 consecutive THR procedures (171 BFX acetabular cups and six CFX acetabular cups) in 145 dogs.

The Labrador Retriever (*n* = 37, 25.5%), crossbred dogs (*n* = 31, 21.4%), German Shepherd dogs (*n* = 21, 14.5%), and Border Collies (*n* = 15, 10.3%) were the four most common breeds, with 10 or fewer of 19 other breeds included in the dataset. There were 84 males (29 neutered) and 61 females (30 neutered). The median bodyweight was 28.90 kg (IQR: 23.0–35.8 kg) and the median age at the time of surgery was 1.9 years (IQR: 0.8–4.5 years). The reason for THR was hip dysplasia (*n* = 134, 92.4%), coxofemoral luxation (*n* = 9, 6.2%), avascular necrosis (n = 1, .7%), and capital physeal fracture (*n* = 1, .7%). There were 90 right THR procedures (50.8%) and 87 left THR procedures (49.2%).

### 3.2 Intra‐ and postoperative complications

The median time to radiographic follow‐up was 88 days (IQR: 84–92 days). The total intra‐ and postoperative complication rate was 24/177 (13.6%). Of these, 9/177 (5.1%) were intraoperative complications and 16/177 (9.0%) were postoperative complications; one THR procedure had both an intra‐ and postoperative complication (intraoperative femoral fissure progressing to postoperative femoral fracture). Of total intra‐ and postoperative complications, 11/177 (6.2%) were stem complications. One THR procedure had both a stem complication (intraoperative femoral fissure) and non‐stem complication (prosthetic luxation not attributable to the stem) with a subsidence value of .1 mm. Of the 14 non‐stem complications, 12 major complications were cup‐related (8 prosthetic luxation and 4 aseptic loosening). There were no prosthetic luxations attributable to the stem. One other major complication (periprosthetic cup surgical site infection) and one minor complication (seroma) were diagnosed. Of the 11 stem complications, 10 were major and one was catastrophic. Eight of the stem complications were intraoperative femoral fissures identified and stabilized with cerclage intraoperatively, 7/8 of these intraoperative femoral fissures did not progress to femoral fracture during the study period. The remaining 1/8 intraoperative femoral fissure progressed to a catastrophic postoperative femoral fracture requiring hindquarter amputation. This complication occurred 21 weeks postoperatively and a subsidence value of 3.0 mm was recorded at 12 weeks postoperatively. There was one intraoperative femoral fracture stabilized with a locking plate and screws. There were two postoperative femoral fractures occurring at 15 and 24 days postoperatively which were stabilized with a locking plate and screws.

**FIGURE 1:**
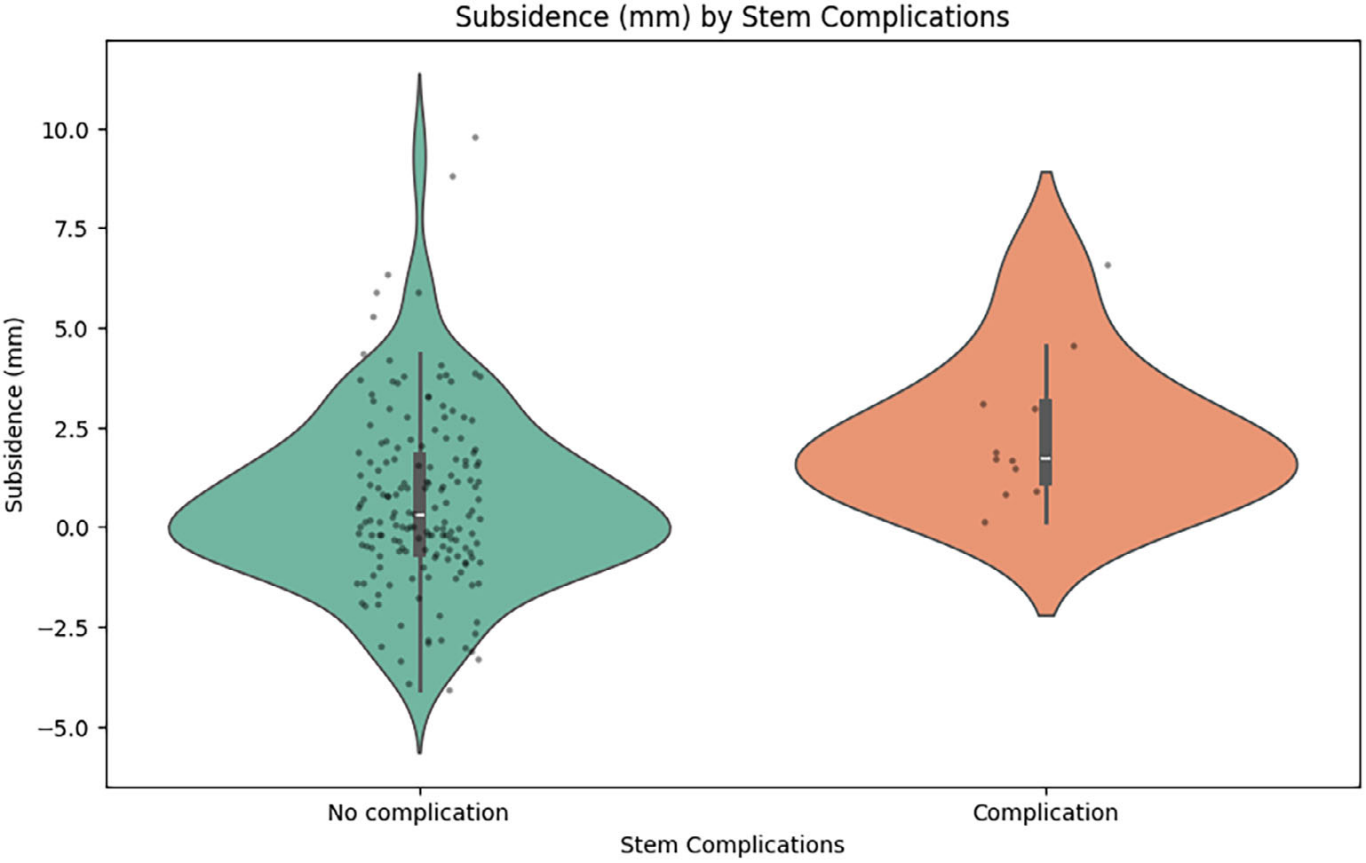
Violin plot showing the distribution of subsidence of the BFX standard stem in dogs with and without stem complications.

Median subsidence across all BFX standard stems at the time of radiographic follow‐up was .4 mm (IQR: −.5–1.9 mm) with a range of −4.1 to 9.8 mm. Subsidence was associated with a stem complication (*p* = .005; Table 1). Subsidence was not associated with a non‐stem complication (*p* = .76) or prosthetic luxation specifically (*p* = .07). Subsidence alone was poorly predictive of a stem complication, with a peak Youden's J‐statistic of .48 at a subsidence value of .7 mm, representing weak‐to‐moderate utility as an indicator of a complication. There were seven stems with a subsidence value of more than 5.0 mm, of these only 1/7 had a stem complication. There were 26 stems with a subsidence value of more than 3.0 mm, of these only 3/26 had a stem complication. There were two stems with large subsidence values of 8.8 and 9.8 mm without a stem complication or any complication. In these two stems, there was no evidence of stem undersizing with CF_cor_ of 74% and 72%, and CF_sag_ of 71% and 61% respectively, and on postoperative templating assessment. There was significant overlap of subsidence values between stems with and without stem complications (Figure 1). There was no association between subsidence and any femoral or stem measurements (Table 2). Six stems had a CF_cor_ greater than 85% and one stem had a CF_sag_ greater than 85%. A total of 18 femora (10.2%) had a CFI ≤1.8. Of the 26 stems with subsidence values of more than 3.0 mm, three stems rotated into retroversion on follow‐up radiographs. These stems had subsidence values of 3.1, 6.6, and 9.8 mm.

**TABLE 1:** Subsidence values of BFX standard stems with and without complications.

Complication type		Subsidence	*p*‐value
Stem complication	Present (*n* = 11)	1.71 mm (IQR: 1.18–3.04 mm) Range: 0.11 mm to 6.59 mm	.**005**
	Absent (*n* = 166)	0.28 mm (IQR: −0.59 ‐ 1.73 mm) Range: −4.07 mm to 9.79 mm	
Non‐stem complication	Present (*n* = 14)	0.80 mm (IQR: −1.13 ‐ 2.76 mm) Range: −2.22 mm to 4.34 mm	.76
	Absent (*n* = 163)	0.37 mm (IQR: −0.53 ‐ 1.76 mm) Range: −4.07 mm to 9.79 mm	
Prosthetic luxation	Present (*n* = 8)	2.10 mm (IQR: 0.55–3.92 mm) Range: −0.89 mm to 4.34 mm	.07
	Absent (*n* = 169)	0.36 mm (IQR: −0.56 ‐ 1.73 mm) Range: −4.07 mm to 9.79 mm	

Abbreviation: IQR, interquartile range.

**TABLE 2:** Univariable testing of femoral and stem measurements against subsidence. Normally distributed measurements reported as mean and standard deviation.

Femoral measurement	Stem complication	No stem complication	*p*‐value
CFI	2.08 (IQR: 1.90–2.33)	2.00 (IQR: 1.90–2.08)	.1264
CF_cor_	73.4% ± 6.1%	76.6% ± 7.9%	.2130
CF_sag_	68.3% ± 7.0%	66.7% ± 8.3%	.5402
SO_cor_	1.9° (IQR: 1.0°–2.9°)	2.1° (IQR: 0.9°–2.6°)	.7128
SO_sag_	1.8° (IQR: 0.6°–3.1°)	1.8° (IQR: 0.7°–3.1°)	.9322

*Note*: Non‐normally distributed measurements reported as median and interquartile ranges.

Abbreviations: CFI, canal flare index; CF_cor_, coronal canal fill; CF_sag_, sagittal canal fill; IQR, interquartile range; SO_cor_, stem orientation coronal; SO_sag_, stem orientation sagittal.

## 4 DISCUSSION

This study reports postoperative subsidence in a large case series of BFX standard stems and examines its association with surgical complications as well as femoral and stem measurements. Subsidence was associated with stem complications but not with other complications, and no femoral or stem measurement predicted subsidence. A wide range of subsidence values was observed, and previously proposed thresholds of 3.0 mm or 5.0 mm were poorly discriminatory for complications.

The association between subsidence and complications may not be a causal association. Most stem complications (8/11) were intraoperative femoral fissures, only one of which progressed to a postoperative femoral fracture. In these cases, the fissure preceded the development of subsidence, suggesting the possibility of reverse causality and that intraoperative fissures predispose to subsidence rather than subsidence causing complications. How a fissure predisposes to subsidence is unknown and may be either due to compromised stability of the cementless stem or through how a fissure affects femoral broaching and impaction of the stem. However, the retrospective nature of this study cannot show a definitive causal relationship between intraoperative fissures and subsidence. This aligns with prior reports that intraoperative fissures are not strongly predictive of postoperative femoral fracture.^11^, ^30^ Our findings are consistent with previous observations that subsidence does not necessarily lead to adverse outcomes. For example, although Mitchell and others^13^ demonstrated that the BFX lateral bolt stem exhibited greater resistance to subsidence than the BFX collared and BFX standard stems, complication rates did not differ between designs. Similarly, Townsend and others^18^ documented up to 15.5 mm of subsidence in a case without subsequent complications. Although subsidence has been proposed to increase fracture risk due to the generation of hoop stresses, the small number of fractures in our study precluded robust statistical analysis.^31^ Townsend and others^18^ reported an association between subsidence and postoperative fracture, but their analysis was based on only three events and may reflect a type I error. Subsidence has also been associated with changes in stem version, with the stem rotating into retroversion.^14^ In this study, stems experiencing more than 3.0 mm of subsidence rarely had significant version changes, although further studies may be useful to determine how proximodistal and rotational stability may be related. Femoral fracture itself also complicates the assessment of subsidence, as stem‐femur disruption can prevent assessment of subsidence.^10^, ^11^ In our study, three cases experienced a catastrophic complication before the minimum follow‐up period for inclusion. Measurement of subsidence may be less reliable in these cases, particularly with femoral fracture where there is derangement of the stem and femur. However, less than 1.0 mm of subsidence was recorded in these cases at the last radiographic follow‐up, and despite not meeting minimum radiographic follow‐up, there was no evidence to implicate subsidence as a contributing factor in any of these cases.

Previous studies have proposed thresholds to define excessive subsidence, most commonly 3.0 mm^13^, ^18^ or 5.0 mm.^9^ However, these cutoffs were not empirically derived and appear to reflect subjective judgments rather than outcome‐based analyses. In our study, both thresholds were poorly discriminatory for complications. Most stems that subsided beyond 3.0 mm or 5.0 mm did not experience either intra‐ or postoperative complications. This suggests that a cutoff may be of limited clinical value. Instead, the clinical relevance of subsidence should be interpreted in the context of other factors including the presence of intraoperative femoral fissures, femoral morphology, implant sizing, and postoperative activity as previously emphasized.^18^

Design modifications such as the BFX lateral bolt and BFX collared stems have been proposed as an alternative to the BFX standard stem for providing a safety cushion in the face of postoperative subsidence.^9^, ^13^ Our findings provide perspective on their use. Although a femoral fissure can predispose to greater magnitudes of subsidence, this migration was almost always clinically inconsequential. The BFX lateral bolt and BFX collared stems require the same femoral preparation as the BFX standard stem and therefore, some postoperative subsidence is to be expected with any BFX stem. The design modifications with the BFX lateral bolt and BFX collared stems may be counterproductive if natural subsidence is prevented which may lead to subsequent toggling of the stem and impaired osseointegration. Parallels can be drawn with the human THR literature. Meta‐analyses have consistently shown that collared cementless stems resist subsidence more effectively than collarless stems, yet revision rates remain comparable between groups.^32^, ^33^ This has led some to question whether subsidence itself is clinically meaningful.^34^ Consistent with these observations, our study demonstrates that revision rates for the BFX standard stem are similar to those previously reported,^4^, ^18^ despite a wide range of observed subsidence magnitudes.

Most canal fill values in this study fell below the traditionally recommended 85% threshold, yet canal fill was not associated with postoperative subsidence. This aligns with prior reports^13^, ^18^ and adds to the growing evidence that the 85% recommendation may be overly conservative. Similarly, no association was identified between CFI and subsidence, consistent with the findings of Mitchell and others.^13^ Notably, only about 10% of femora in our study had a CFI ≤1.8, which may reflect a population bias in this single‐institutional retrospective study. Notably, there was no evidence of surgeon selection bias as only two dogs eligible for a BFX stem were selected for CFX stem implantation. Although varus stem orientation has previously been linked to an increased risk of intraoperative femoral fissure,^18^ this association was not observed here.

The broader characteristics of our cohort were comparable to those in other reports. Case selection, implant sizing, and complication rates were similar across studies, and the demographics of dogs undergoing THR were broadly consistent with previous descriptions.^1^, ^10^ The magnitude of subsidence observed also fell within the ranges previously reported.^10^, ^13^, ^18^ Although our sample population exhibited slightly higher CFI values than some prior series,^10^, ^13^ this again may reflect a case selection effect. Finally, complication rates and the types of complications were similar to those documented in other studies.^4^, ^18^

This study had several limitations. Its retrospective, single‐institution design and the fact that all THR procedures were performed by a single surgeon may introduce biases that limit generalizability. We did not include a comparator group of BFX lateral bolt or BFX collared stems, which restricts direct assessment of implant design differences. Although this represents the largest non‐registry case series of the BFX standard stem to date, the rarity of certain complications, particularly postoperative femoral fractures, limited the power of our analyses. Measurement error is also possible due to variation in radiographic positioning,^21^ although the distribution of negative values was comparable to previous reports.^10^, ^13^ Proximal migration of the stem is impossible and therefore negative subsidence values reflect variation in radiographic positioning. Given that negative subsidence values are seen across multiple studies utilizing plain radiography, computed tomography should be strongly encouraged in future studies to more accurately measure postoperative subsidence.

In summary, subsidence of the BFX standard stem was associated with stem complications, most of which were intraoperative femoral fissures. Subsidence was not associated with non‐stem complications, and substantial subsidence may occur without adverse outcomes. All four catastrophic complications had subsidence values of 3.0 mm or less. Previously suggested thresholds of 3 mm and 5 mm were not useful in predicting complications. Further studies are needed to clarify when subsidence is clinically important.

## AUTHOR CONTRIBUTIONS

Low D, BVetMed, CertAVP (GSAS), PGCertVPS, MRCVS: Study design, data collection, data analysis, interpretation of results, and manuscript writing. Treharne R, BVetMed, PGCertSAS, MRCVS: Study design, data collection, interpretation of results, and manuscript writing. Rutherford S, BVMS, CertSAS, DipECVS, FRCVS: Study conception, study design, interpretation of results, and manuscript revision. All authors approved the final manuscript.

## FUNDING INFORMATION

No funding or grants to declare.

## CONFLICT OF INTEREST STATEMENT

No conflicts of interest to declare.

## References

[1] Marcellin‐Little DJ , De Young BA , Doyens DH , De Dyoung DJ . Canine Uncemented porous‐coated anatomic total hip arthroplasty: results of a long‐term prospective evaluation of 50 consecutive cases. Vet Surg. 1999;28(1):10‐20. doi:10.1053/jvet.1999.0010 10025635

[2] Gemmill TJ , Pink J , Renwick A , et al. Hybrid cemented/cementless total hip replacement in dogs: seventy‐eight consecutive joint replacements. Vet Surg. 2011;40(5):621‐630. doi:10.1111/j.1532-950X.2011.00827.x 21521239

[3] Bergh MS , Budsberg SC . A systematic review of the literature describing the efficacy of surgical treatments for canine hip dysplasia (1948–2012). Vet Surg. 2014;43(5):501‐506. doi:10.1111/j.1532-950X.2014.12208.x 24837650

[4] Allaith S , Tucker LJ , Innes JF , et al. Outcomes and complications reported from a multiuser canine hip replacement registry over a 10‐year period. Vet Surg. 2023;52(2):196‐208. doi:10.1111/vsu.13885 36062338 PMC10087566

[5] Forster KE , Wills A , Torrington AM , et al. Complications and owner assessment of canine total hip replacement: a multicenter internet based survey. Vet Surg. 2012;41(5):545‐550. doi:10.1111/j.1532-950X.2012.01015.x 22731937

[6] Henderson ER , Wills A , Torrington AM , et al. Evaluation of variables influencing success and complication rates in canine total hip replacement: results from the British veterinary Orthopaedic association canine hip registry (collation of data: 2010–2012). Vet Rec. 2017;181(1):18‐18. doi:10.1136/vr.104036 28386028

[7] Ota J , Cook JL , Lewis DD , et al. Short‐term aseptic loosening of the femoral component in canine total hip replacement: effects of cementing technique on cement mantle grade. Vet Surg. 2005;34(4):345‐352. doi:10.1111/j.1532-950X.2005.00053.x 16212589

[8] Edwards MR , Egger EL , Schwarz PD . Aseptic loosening of the femoral implant after cemented total hip arthroplasty in dogs: 11 cases in 10 dogs (1991‐1995). J Am Vet Med Assoc. 1997;211(5):580‐586. 9290824

[9] Liska WD , Doyle ND . Use of an electron beam melting manufactured titanium collared cementless femoral stem to resist subsidence after canine total hip replacement. Vet Surg. 2015;44(7):883‐894. doi:10.1111/vsu.12353 26138323

[10] Kokkinos P , Parsons K , Belch A , Barthelemy N . The influence of age at total hip replacement on perioperative complications associated with a press‐fit cementless stem with lateral bolt in dogs. Vet Surg. 2025;54(3):581‐593. doi:10.1111/vsu.14203 39815446 PMC11947299

[11] Ganz SM , Jackson J , VanEnkevort B . Risk factors for femoral fracture after canine press‐fit cementless total hip arthroplasty. Vet Surg. 2010;39(6):688‐695. doi:10.1111/j.1532-950X.2010.00694.x 20459487

[12] Nelson LL , Dyce J , Shott S . Risk factors for ventral luxation in canine total hip replacement. Vet Surg. 2007;36(7):644‐653. doi:10.1111/j.1532-950X.2007.00316.x 17894590

[13] Mitchell MM , Hudson CC , Beale BS . Comparison of femoral stem subsidence between three types of press‐fit cementless total hip replacement in dogs. Vet Surg. 2020;49(4):787‐793. doi:10.1111/vsu.13391 32086832

[14] Israel SK , Liska WD . Outcome of canine cementless collared stem total hip replacement with proximal femoral periprosthetic cerclage application: 184 consecutive cases. Vet Surg. 2022;51(2):270‐278. doi:10.1111/vsu.13740 34655241

[15] Leiss F , Götz JS , Meyer M , et al. Differences in femoral component subsidence rate after THA using an uncemented collarless femoral stem: full weight‐bearing with an enhanced recovery rehabilitation versus partial weight‐bearing. Arch Orthop Trauma Surg. 2022;142(4):673‐680. doi:10.1007/s00402-021-03913-0 34019145 PMC8924083

[16] Bornes TD , Radomski LR , Bonello JP , et al. Subsidence of a single‐taper femoral stem in primary total hip arthroplasty: characterization, associated factors, and sequelae. J Arthroplasty. 2023;38(7):S174‐S178. doi:10.1016/j.arth.2023.04.026 37088226

[17] Buks Y , Wendelburg KL , Stover SM , Garcia‐Nolen TC . The effects of interlocking a universal hip cementless stem on implant subsidence and mechanical properties of cadaveric canine femora. Vet Surg. 2016;45(2):155‐164. doi:10.1111/vsu.12437 26767439 PMC5066748

[18] Townsend S , Kim SE , Pozzi A . Effect of stem sizing and position on short‐term complications with canine press fit cementless total hip arthroplasty. Vet Surg. 2017;46(6):803‐811. doi:10.1111/vsu.12666 28460422

[19] BioMedtrix Inc . Canine Modular Total Hip Replacement System, Surgical Protocol for BFXTM Cementless Application. 2008.

[20] Cook JL , Evans R , Conzemius MG , et al. Proposed definitions and criteria for reporting time frame, outcome, and complications for clinical orthopedic studies in veterinary medicine. Vet Surg. 2010;39(8):905‐908. doi:10.1111/j.1532-950X.2010.00763.x 21133952

[21] Brand KJ , Beale BS , Hudson CC . Evaluation of a novel method to calculate cementless femoral stem level on craniocaudal projection radiographs. Vet Surg. 2021;50(8):1592‐1599. doi:10.1111/vsu.13723 34545581

[22] Rashmir‐Raven AM , DeYOUNG DJ , Abrams CF , Aberman HA , Richardson DC . Subsidence of an uncemented canine femoral stem. Vet Surg. 1992;21(5):327‐331. doi:10.1111/j.1532-950X.1992.tb01705.x 1413463

[23] Korani HM , Marcellin‐Little DJ , Roe SC . Variability associated with assessing changes in position of a canine uncemented femoral stem prosthesis. Vet Comp Orthop Traumatol. 2015;28:409‐416. doi:10.3415/VCOT-15-03-0044 26449547

[24] McKinney W . Data structures for statistical computing in Python. Python in Science Conference; 2010:56‐61. doi:10.25080/Majora-92bf1922-00a

[25] Harris CR , Millman KJ , Van Der Walt SJ , et al. Array programming with NumPy. Nature. 2020;585(7825):357‐362. doi:10.1038/s41586-020-2649-2 32939066 PMC7759461

[26] Virtanen P , Gommers R , Oliphant TE , et al. SciPy 1.0: fundamental algorithms for scientific computing in python. Nat Methods. 2020;17(3):261‐272. doi:10.1038/s41592-019-0686-2 32015543 PMC7056644

[27] Pedregosa F , Varoquaux G , Gramfort A , et al. Scikit‐Learn: Machine Learning in Python. 2012;12:2825‐2830. doi:10.48550/ARXIV.1201.0490

[28] Waskom M . seaborn: statistical data visualization. J Open Source Softw. 2021;6(60):3021. doi:10.21105/joss.03021

[29] Hunter JD . Matplotlib: A 2D graphics environment. Comput Sci Eng. 2007;9(3):90‐95. doi:10.1109/MCSE.2007.55

[30] Kwok JY , Wendelburg KL . Clinical outcomes of canine total hip replacement utilizing a BFX lateral bolt femoral stem: 195 consecutive cases (2013–2019). Vet Surg. 2023;52(1):51‐61. doi:10.1111/vsu.13871 36181274

[31] McCulloch RS , Roe SC , Marcellin‐Little DJ , Mente PL . Resistance to subsidence of an uncemented femoral stem after cerclage wiring of a fissure. Vet Surg. 2012;41(1):163‐167. doi:10.1111/j.1532-950X.2011.00858.x 21770982

[32] Nerys‐Figueroa J , Parsa A , Curley A , Charif S , Domb BG , Schinsky MF . Slightly reduced early subsidence with similar outcomes and complications rate in collared stems ‐ a systematic review of randomized clinical trials. J Orthop. 2024;50:170‐176. doi:10.1016/j.jor.2024.01.013 38328796 PMC10845209

[33] Watanabe R , Mishima H , Totsuka S , Nishino T , Yamazaki M . Primary stability of collared and collarless cementless femoral stems – a finite element analysis study. Arthroplasty Today. 2023;21:101140. doi:10.1016/j.artd.2023.101140 37151402 PMC10160691

[34] Giovanoulis V , Kenanidis E , Aïm F , et al. Collared versus collarless hydroxyapatite‐coated stems for primary cementless total hip arthroplasty; a systematic review of comparative studies. Is there any difference in survival, functional, and radiographic outcomes? SICOT‐J. 2024;10:8. doi:10.1051/sicotj/2024003 38358293 PMC10868518

